# Neuroscience: genes, brains, and reading variability

**DOI:** 10.1038/s44271-023-00025-w

**Published:** 2023-10-03

**Authors:** Saloni Krishnan, Sonia Singh

**Affiliations:** Royal Holloway, University of London, London, UK

## Abstract

New research demonstrates the use of large-scale datasets to study the role of environmental and biological factors in reading ability.

**Figure:**
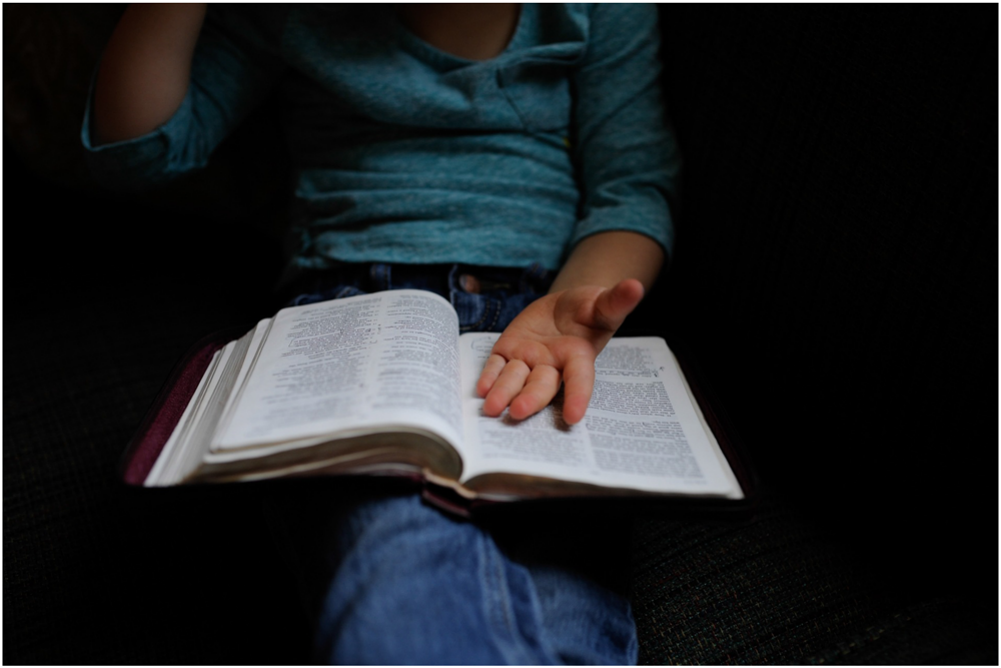
StockSnap on Pixabay

Reading involves transforming arbitrary visual symbols into sounds to unlock meaning. This skill is fundamental in early education. Reading challenges can severely affect economic, social and health outcomes. While access to high-quality reading instruction and environments is undeniably important, a crucial element of developing effective support and intervention is an understanding of the biological contributors to reading ability. Does brain structure influence reading ability? How do genes contribute?

Carrión-Castillo and colleagues at the Basque Center on Cognition, Brain and Language addressed these questions using the expansive ABCD dataset^1^. The researchers interrogated how reading performance is related to brain structure in 9000+ American children aged 9–10, focusing on cortical surface area and cortical thickness (measures with distinct developmental trajectories and genetic influences). Their main outcome measure, word reading, was correlated with total cortical surface area, as well as eight other regional measures, including the cortical surface area of the left lateral superior temporal gyrus and cortical thickness in occipital regions.

Owing to the scale of their sample, Carrión-Castillo and colleagues could interrogate the extent to which genes contribute to brain-reading associations using polygenic risk scores. This offers a significant advance over smaller candidate gene studies. The authors found genes contributing to word reading performance were linked with cortical surface area in these regions, suggesting these genes contributed to both brain structure and poor behaviour. Observed associations between genetics and behaviour, and the brain and genetics, were small. This suggests these relationships are complex, however, the variability explained is likely to improve with further data collection for genome-wide association studies.

The focus of this work was on word reading, not other aspects of reading such as comprehension or decoding. Further, the influence of problems of language or attention, which frequently co-occur with reading challenges, was not assessed. Improving the usability of large-scale imaging data (for instance, enabling digital collection of new behavioural data from participants to develop more nuanced phenotypes) would help to address these challenges.

Altogether, this demonstrates the importance of large-scale datasets, allowing us to understand complex links between genes, brains, and behaviour. We need population neuroscience efforts across languages, cultures, and educational contexts to further understand these complex interactions, ideally using newer sensitive, nuanced neural measures.
